# Markers of Coagulation and Fibrinolysis Predicting the Outcome of Acute Ischemic Stroke Thrombolysis Treatment: A Review of the Literature

**DOI:** 10.3389/fneur.2019.00513

**Published:** 2019-06-21

**Authors:** Zsuzsa Bagoly, István Szegedi, Rita Kálmándi, Noémi Klára Tóth, László Csiba

**Affiliations:** ^1^Division of Clinical Laboratory Sciences, Department of Laboratory Medicine, Faculty of Medicine, University of Debrecen, Debrecen, Hungary; ^2^MTA-DE Cerebrovascular and Neurodegenerative Research Group, University of Debrecen, Debrecen, Hungary; ^3^Department of Neurology, Clinical Centre, University of Debrecen, Debrecen, Hungary

**Keywords:** thrombolysis, coagulation, fibrinolysis, stroke, outcome

## Abstract

Intravenous administration of recombinant tissue plasminogen activator (rt-PA) has been proven to be safe and effective in the treatment of acute ischemic stroke. Little is known, however, why this treatment is less effective in some patients while in others life-threatening side-effects, e.g., symptomatic intracerebral hemorrhage might occur. Clinical failure of thrombolysis related to absent or partial recanalization or reocclusion as well as hemorrhagic complications of thrombolysis are possibly related to hemostatic events. Data on markers of coagulation and/or fibrinolysis in acute stroke patients are numerous and may provide indications regarding therapy outcomes. Better understanding of the hemostatic and fibrinolytic system during rt-PA therapy might be clinically useful and ultimately might lead to an improvement in the efficacy or safety of this treatment. Studies on thrombus composition retrieved from cerebral arteries may also advance our knowledge and provide a key to improve acute stroke therapy. Here we provide a comprehensive review on a wide range of factors and markers of coagulation and fibrinolysis that have been studied in the context of thrombolysis outcome in ischemic stroke patients. Moreover, a brief summary is given on the most recent research on thrombus composition having a potential influence on outcomes.

## Introduction

Ischemic stroke is one of the most important and serious vascular diseases affecting millions of people all over the world (1). By causing paresis, gait disturbance, speech disturbances and other symptoms stroke increases morbidity and mortality significantly (2). Despite the emerging importance of endovascular stroke therapy, e.g., mechanical thrombectomy (3), the primary treatment of acute ischemic stroke today is thrombolysis using recombinant tissue plasminogen activator within the therapeutic time window. The 1995 National Institute of Neurological Disorders and Stroke rt-PA (recombinant tissue plasminogen activator) Stroke Study (NINDS trial) was a milestone for stroke therapy as it proved that treating ischemic stroke patients with rt-PA within 3 h of the onset of stroke symptoms results in a considerable neurological improvement at 3 months as compared to the placebo group without significant change in mortality (4). The ECASS (European Cooperative Acute Stroke Study) III trial showed that intravenous rt-PA treatment is efficient beyond the time window of 3 h as compared to placebo in a certain group of ischemic stroke patients (5). By extending the time window to up to 4.5 h, 52.4% of patients had a favorable outcome as compared to the placebo group. Despite the unquestionable effectiveness of rt-PA in the treatment of acute ischemic stroke, this treatment is not a remedy for all. The rate of early recanalization using rt-PA is ~25% in patients with a proximal middle cerebral artery occlusion and only 10% in patients with an internal carotid occlusion (6). Additionally, the rate of re-occlusion is as high as 30% (7). Besides recanalization failure, about 6–8% of patients develop intracranial hemorrhage as side-effect, despite taking all precautionary steps to minimize bleeding risk (8). Little is known why in some patients recanalization failure occurs while in others bleeding will take place. In theory, the effect of rt-PA might depend on hemostasis factors affecting clot structure. The aim of this review article is to provide an insight on coagulation and fibrinolysis parameters that can have an impact on or were shown to have an association with the outcome of thrombolysis in ischemic stroke patients. Recent studies on thrombus composition obtained via endovascular treatment from the cerebral arteries of ischemic stroke patients are also summarized.

## Brief Overview of the Non-hemostasis Factors Affecting Thrombolysis Outcome

Although the Safe Implementation of Thrombolysis in Stroke-MOnitoring STudy (SITS-MOST) proved the efficiency and safety of intravenous rt-PA (8), our understanding of predictive factors affecting thrombolysis outcomes is still limited. Poor outcome of thrombolysis is generally defined at 3 months post-event as a modified Rankin score (mRS) of 3 or more (less frequently 2 or more), which includes mortality (mRS = 6) (9). Thrombolytic therapy-related hemorrhage has been defined by the NINDS, ECASS I, ECASS II, ECASS III, and the SITS-MOST studies. (10) Symptomatic intracranial hemorrhage (sICH) was defined as any neurological deterioration associated with hemorrhage within 36 h of rt-PA therapy in the NINDS trial, while in ECASS III and SITS-MOST studies intracranial hemorrhage and ≥4 point increase in baseline NIHSS was considered as sICH [for a comprehensive review, see (10)]. There are several baseline clinical factors which may affect the outcomes of rt-PA therapy including male gender, stroke severity on admission, infarct size, advanced age, hyperglycaemia, etc., nevertheless, due to their low predictive value, these factors are mostly unhelpful for individual treatment decisions (9). Wahlgren et al. adjusted the outcomes of the SITS-MOST to the baseline characteristics of randomized, controlled trials in order to find parameters that can predict the outcome of the thrombolysis (11). In a multivariable analysis it was found that older age, high blood glucose, high National Institutes of Health Stroke Scale (NIHSS) score and definitive ischemic lesion on imaging scans were related to poor outcome (mRS 3–6). Male sex, disability before the actual stroke (modified Rankin Score 2–5), congestive heart failure, diastolic blood pressure, antiplatelet therapy other than aspirin, treatment in new centers were associated with higher mortality at the third month of the follow-up. Atrial fibrillation, systolic blood pressure, and weight were predictors of the occurrence of sICH, but current smokers had a lower rate of sICH.

Among standard baseline laboratory parameters, few were found to be associated with thrombolysis outcomes. In a systematic review based on 54 previous reports unadjusted and adjusted meta-analysis of high admission glucose level showed association with poor outcome (mRS≥2) and sICH (12). In a relatively large study, admission neutrophil count and neutrophil to lymphocyte ratio was found to be independently associated with increased risk of sICH and worse outcomes (mRS≥3) at 3 months (13).

The disruption of blood-brain barrier (BBB) is thought to be associated with post-lysis ICH (14). Early detection of BBB changes using imaging techniques (contrast-enhanced MRI or CT) are promising tools to predict hemorrhagic transformation (15, 16). The emerging role of imaging techniques in identifying therapy failure and safety is out of the scope of this review, interested readers should consult recent publications (17, 18).

Unfortunately, despite all efforts to predict patient subgroups that are more likely to clearly benefit or not benefit from intravenous thrombolysis, it is not yet possible to predict individual outcomes infallibly based on the above listed baseline clinical, standard laboratory or imaging variables (19). Moreover, known predictors of worse functional outcome due to inefficacy of therapy and the predictors of symptomatic intracranial hemorrhage show an overlap. This suggests a potential role for other factors in the diverse pathomechanisms ultimately leading to poor clinical outcomes.

**Figure 1:**
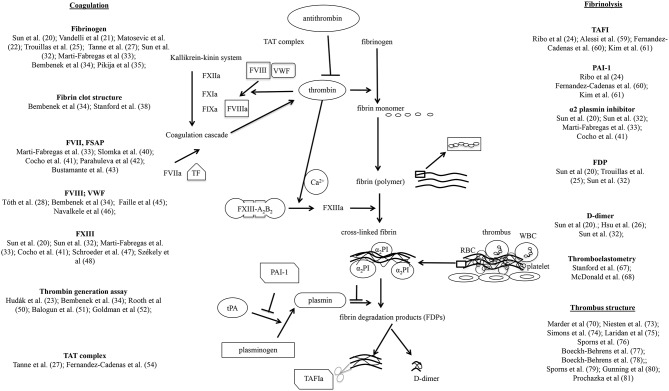
Simplified scheme demonstrating the main elements of coagulation, fibrinolysis, and thrombus formation, depicting publications that investigated the relationship of these elements with the outcome of thrombolytic therapy applied in acute ischemic stroke. α2PI, α2 plasmin inhibitor; FVII, factor VII; FVIIa, activated factor VII; FVIII, factor VIII; FVIIIa, activated factor VIII; FIXa, activated factor IX; FXIa, activated factor XI; FXIIa, activated factor XII; FXIII, factor XIII; FXIIIa, activated factor XIII; FXIII-A_2_B_2_, plasma (tetramer) form of factor XIII; PAI-1, plasminogen activator inhibitor-1; RBC, red blood cell; TAFI, thrombin-activatable fibrinolysis inhibitor TAFIa, activated thrombin-activatable fibrinolysis inhibitor; TAT complex, thrombin-antithrombin complex; TF, tissue factor; tPA, tissue plasminogen activator; VWF, von Willebrand Factor; WBC, white blood cell.

Here we give a comprehensive list of hemostasis parameters that were reported to be associated with thrombolysis outcomes. Hemostasis parameters showing significant association with hemorrhagic transformation after thrombolysis are summarized in Table 1, while markers with a significant association regarding poor outcome based on mRS are listed in Table 2. A simplified overview of the coagulation and fibrinolytic system and cited literature investigating the potential role of certain factors/markers in the outcome of thrombolytic therapy is provided on Figure 1.

**Table 1:** Hemostasis markers associated with hemorrhagic transformation after acute ischemic stroke thrombolysis.

**Predictive marker**	**References**	**Definition of ICH**	**Time of blood collection**	**Cut-off value**	**OR**	**95% CI**	***P***	**ICH/Total patient cohort**	**aSICH/SICH**	**Association with outcome**
Fibrinogen	Sun et al. (20)	ECASS I	2 h post-lysis	<2 g/L	12.82	1.13–145.80	0.04	17 (6 ePH and 11 eHI)/72	n.a.	Higher risk of ePH
Fibrinogen	Vandelli et al. (21)	NINDS	2 h post-lysis	<2 g/L and/or >25% decrease	7.43	2.620–21.100	<0.001	24/104	18/6	Higher risk of ICH
Fibrinogen	Matosevic et al. (22)	NINDS	6 h post-lysis	decrease of ≥2 g/L (Δ fibrinogen 0–6 h)	4.53	2.39–8.60	<0.0001	47/547	14/33	Higher risk of SICH and major systemic bleeding
ETP	Hudák et al. (23)	ECASS II	Before thrombolysis	<1265.9 nM x min	17.54	1.45–212.72	<0.05	13/120	7/6	Higher risk of SICH
Peak thrombin	Hudák et al. (23)	ECASS II	Before thrombolysis	<204.7 nM	15.12	1.38–166.02	<0.05	13/120	7/6	Higher risk of SICH
PAI-1	Ribo et al. (24)	ECASS I	Before thrombolysis	<21.4 ng/mL	12.75	1.17–139.2	0.04	17/77	11/6	Higher risk of SICH
TAFI	Ribo et al. (24)	ECASS I	Before thrombolysis	>180%	12.9	1.41–118.8	0.02	17/77	11/6	Higher risk of SICH
FDP	Trouillas et al. (25)	ECASS I	2 h post-lysis	Increase by >200 mg/L as compared to baseline	4.95	1.09–22.4	0.03	42 (11 ePH and 31 eHI) /157	n.a.	Higher risk of ePH
FDP	Sun et al. (20)	ECASS I	2 h post-lysis	n.a.^*^	7.50	1.26-44.61	0.03	17 (6 ePH and 11 eHI)/72	n.a.	Higher risk of ePH
D-dimer	Hsu et al. (26)	ECASS II	24 h post-lysis	n.a.^*^	2.97	1.15–7.70	0.025	37/159	31/6	Higher risk of SICH

*Only studies showing significant associations with hemorrhagic transformation after thrombolysis are summarized. aSICH, asymptomatic intracerebral hemorrhage; CI, confidence interval; ECASS, European Cooperative Acute Stroke Study; eHI, early hemorrhagic infarct; ePH, early parenchymal hematoma; ETP, endogenous thrombin potential; FDP, fibrin(ogen) degradation product; ICH, intracerebral hemorrhage; n.a., not available; NINDS, National Institute of Neurological Disorders and Stroke rt-PA (recombinant tissue plasminogen activator) Stroke Study; OR, odds ratio; PAI-1, plasminogen activator inhibitor-1; SICH, symptomatic intracerebral hemorrhage; TAFI, thrombin-activatable fibrinolysis inhibitor*,

* *Per unit change of log transformed data*.

**Table 2:** Hemostasis markers associated with poor outcome at 3 months after acute ischemic stroke thrombolysis.

**Predictive marker**	**References**	**Definition of poor outcome**	**Time of blood collection**	**Cut-off value**	**OR**	**95% CI**	***P***	**Poor outcome/Total patient cohort**
Fibrinogen	Tanne et al. (27)	mRS = 6	24 h post-lysis	>1 g/L as compared to baseline	1.42	1.05–1.91	n.a.	n.a./545
FVIII	Tóth et al. (28)	mRS ≥ 3	Immediately after thrombolysis	>168%	7.10	1.77–28.38	0.006	51/131
			24 h post-lysis	>168%	4.67	1.42–15.38	0.011	
VWF	Tóth et al. (28)	mRS ≥ 3	Immediately after thrombolysis	>160%	6.31	1.83–21.73	0.003	51/131
			24 h post-lysis	>160%	19.02	1.94–186.99	0.012	
ETP	Hudák et al. (23)	mRS = 6	Before thrombolysis	1265.9 nM x min	5.28	1.27–21.86	< 0.05	26/120
TAT complex	Tanne et al. (27)	mRS = 6	24 h post-lysis	n.a.^*^	1.72	1.26–2.34	0.0006	n.a./361
D-dimer	Hsu et al. (26)	mRS ≥ 3	After initiation of thrombolysis within 24 h after stroke onset	n.a.^*^	1.90	1.27–2.86	0.002	79/159

*Only studies showing significant associations with poor outcome after thrombolysis are summarized. CI, confidence interval; ETP, endogenous thrombin potential; FVIII, factor VIII; mRS, modified Rankin Scale; n.a., not available; OR, odds ratio; VWF, von Willebrand Factor; TAT complex, thrombin-antithrombin complex*,

* *per unit change of log transformed data*.

## Coagulation Factors/Markers Of Coagulation Activation Associated With Thrombolysis Outcomes

### Fibrinogen

Fibrinogen is a central protein of hemostasis, although it has considerable non-hemostasis related functions as well: it plays a role in cellular and matrix interactions, inflammatory response, wound healing, cancer progression (29). Fibrinogen is the substrate of the procoagulant enzyme thrombin that cleaves it into fibrin. Fibrin forms a branched three-dimensional network that is cross-linked by activated factor XIII (FXIIIa) in the last step of the coagulation cascade (30). Cross-linked fibrin forms a network providing a stable biochemical and biophysical support to blood clots. Fibrin acts as a cofactor in tPA-induced plasminogen activation thus linking fibrin formation and fibrinolysis. Variations in fibrin properties and fibrin clot structure ultimately affect its degradation by plasmin (29, 31).

Low circulating fibrinogen levels (or a significant early decrease in fibrinogen levels after therapy) have been associated with post-thrombolysis hemorrhage in ischemic stroke patients (Table 1), although some studies showed contradictory results. In 2004, the phenomena of “early fibrinogen degradation coagulopathy” was proposed, describing a biological syndrome predictive of cerebral bleeding post-thrombolysis related to an early loss of fibrinogen and thus characterized by the increase of fibrin(ogen) degradation products (FDPs) (25). These results were later confirmed by other studies, including that of Sun et al. (20) (Table 1). In that study, 72 consecutive ischemic stroke patients treated with rt-PA were enrolled, and it was shown that a decrease in fibrinogen levels at 2 h post-lysis to <2 g/l multiplies the odds of early parenchymal hemorrhage by a factor of 12.82. It has to be noted, however, that in another study by the same authors somewhat different conclusions were reached (32). In that study 80 consecutive ischemic stroke patients were involved and changes in coagulation and fibrinolytic parameters were studied after rt-PA induced thrombolysis. Fibrinogen levels showed a 20% mean decrease, which was statistically linked to a decrease in plasminogen levels and it was also related to decreases of factors inhibiting thrombolysis (FXIII, α2-plasmin inhibitor). Nevertheless, fibrinogen levels didn't show a direct correlation with outcomes.

The concept of low fibrinogen levels being associated with post-lysis intracranial hemorrhage was confirmed by two other studies (21, 22) (Table 1). One hundred and four stroke patients, treated with intravenous thrombolysis, were included in the study by Vandelli et al. (21). Fibrinogen levels were measured on admission and 2 h after the thrombolysis. After 7 days of follow-up 24 patients presented with intracranial hemorrhage, of whom 6 patients were symptomatic. Among the 24 patients with intracerebral hemorrhage, 18 belonged to the “low fibrinogen group” (classified as levels decreased to <2 g/L and/or by 25% or more). Multivariate logistic regression analysis confirmed that patients belonging to the “low fibrinogen group” had a significantly increased risk for therapy-associated intracranial hemorrhage (OR: 7.47, 95%CI: 2.26–24.74, *p* < 0.001) and among the conventional risk factors only baseline NIHSS score conferred a statistically significant risk (OR: 1.15, 95%CI: 1.06–1.25, *p* < 0.001). On the other hand, they also demonstrated that higher baseline fibrinogen levels seem to enhance the risk of more significant decrease after thrombolysis. These data are in line with results published by Matosevic et al. who also showed that a reduction of at least 2 g/L in fibrinogen levels at 6 h post-lysis increases the risk of symptomatic intracranial hemorrhage (22). In this study a relatively large (*n* = 547) consecutive stroke patient cohort was investigated, and it was found that quantification of fibrinogen depletion after stroke thrombolysis significantly improved routine risk prediction of bleeding complications.

The association between admission fibrinogen levels and poor outcomes at 3 months post-lysis is not entirely clear. Tanne et al. investigated fibrinogen levels among other hemostasis parameters in patients with acute ischemic stroke from the NINDS rt-PA Stroke Study (27) (Table 2). Fibrinogen levels were measured at baseline, at 2 and 24 h after the thrombolysis. Of the 624 patients of the trial (consisting of rt-PA treatment and placebo group) the plasma samples of 465 patients were available at baseline, after 2 h or after 24 h and in the case of 281 patients all 3 plasma samples were available and were used for further evaluation. Fibrinogen levels showed a decrease at 2 h and remained low after 24 h in the treatment group and the placebo group as well. Higher levels of fibrinogen at baseline were associated with infarct lesion volume as detected by CT at 3 months within the entire study cohort (*n* = 570; *p* = 0.05). Higher 24 h fibrinogen levels were associated with an approximately 40% increase in the odds of death by 90 days in the whole study group (OR: 1.42; 95% CI: 1.05–1.91 per 1 g/L increment), but no other significant associations with outcomes were detected.

On the other hand, in an earlier study by Marti-Fabregas et al. including 83 patients treated by intravenous rt-PA, admission fibrinogen levels showed no association with long-term outcomes as determined by mRS>2 at 3 months (33). Similarly, in a most recent paper, fibrinogen levels on admission, at 24 h and 3 months post-event did not differ significantly in patients with poor outcomes (mRS>2) as compared to those with favorable outcomes at 3 months (34).

The association of fibrinogen levels on admission with imaging results found by Tanne et al. were confirmed in a hyperdense artery study Pikija et al. (35). The authors assessed on-admission fibrinogen level and clot burden in relation with the severity of the stroke, the volume of the infarct and in-hospital mortality in 132 ischemic stroke patients with hyperdense artery sign admitted within 6 h from the onset of the symptoms. Thrombolysis was performed in 60% of the patients and thrombectomy in 44% of them. Increased fibrinogen levels on admission showed association with smaller clot burden and lower NIHSS on admission, while patients with decreased fibrinogen had a higher clot burden and bigger volume of the infarct. However, in the adjusted statistical model, admission fibrinogen levels were not associated significantly with in hospital survival or death.

As conclusion, according to these data assessment of plasma fibrinogen levels pre- and post-lysis could be potentially useful to predict post-lysis intracranial hemorrhage but more data is needed regarding its associations with poor outcomes. It must be noted that determination of fibrinogen levels by the Clauss method is a relatively quick, easy and cheap measurement that can be performed in most clinical laboratories in any hour of the day, which is a clear advantage as compared to many other potentially useful biomarkers. Nevertheless, future studies involving more patients are needed to clearly define its predictive value and to validate its use as a potential biomarker for rt-PA associated bleeding events.

### Fibrin Clot Structure

The structure of the fibrin clots is determined by genetic and environmental factors. Fibrin clots composed of thin, highly branched fibrin fibers are more rigid, less permeable, and less susceptible to dissolution by fibrinolysis (36). It has been shown that patients with ischemic stroke have reduced clot permeability and have features of prothrombotic clot phenotype as compared to healthy individuals (37). On the other hand, changes in clot microstructure of ischemic stroke patients who underwent thrombolysis have been studied scarcely. In a prospective cohort study by Stanford et al. clot microstructure was compared in ischemic stroke patients before, at 2–4 h and at 24 h after thrombolysis to assess the effects of thrombolysis (38). It was shown that thrombolysis itself has an effect on clot structure, but due to the relatively small number of patients who received rt-PA (*n* = 32) no conclusions were reached regarding patient outcomes. In a most recent paper by Bembenek et al. clot properties of 74 ischemic stroke patients undergoing thrombolysis were studied and their impact on clinical outcome was assessed (34). *Ex vivo* plasma fibrin clot formation was investigated on admission, at 24 h and at 3 months post-lysis. Compared with the pretreatment values, fibrin networks assessed at 24 h post-lysis were formed more slowly, were less compact, composed of thinner fibers, which lysed more rapidly. Logistic regression adjusted for potential confounders showed that pretreatment clot lysis time predicted excellent outcome as measured by mRS (0–1). In conclusion, formation of denser fibrin clots that show impaired lysability and the pattern of their changes might be indicative of clinical outcomes in acute stroke patients treated with thrombolysis.

### Factor VII (FVII), Factor VII Activating Protease (FSAP)

The complex of activated factor VII (FVIIa) and tissue factor is the most potent activator that initiates the blood clotting cascade in normal hemostasis (39). FVII levels have been investigated in a handful of studies involving ischemic stroke patients undergoing thrombolysis but no relevant association was found between FVII levels and treatment outcomes or safety (33, 40, 41).

Factor VII activating protease (FSAP)—predominantly expressed in the liver—is a plasma serine protease that activates coagulation factor VII as well as pro-urokinase. Besides, FSAP inactivates tissue factor pathway inhibitor (TFPI), which would have a procoagulant effect on hemostasis as well (42). One hundred and twenty acute stroke patients were prospectively studied by Bustamente et al. in order to determine whether plasma FSAP levels have an association with recanalization after thrombolysis (43). The authors found that lower FSAP antigen levels correlates with a higher chance of recanalization after thrombolysis, suggesting the involvement of FSAP in rt-PA induced clot lysis, however, FSAP levels showed no significant association with poor outcomes (mRS>2) at 3 months post-event.

### Factor VIII (FVIII), Von Willebrand Factor (VWF)

Factor VIII (FVIII), a key component of the blood coagulation system, is a cofactor for factor IXa that converts factor X to the activated form (FXa). In plasma, it circulates with von Willebrand factor (VWF) in a non-covalent complex (44). Associations between FVIII or VWF levels and stroke thrombolysis outcomes were investigated in four recent studies (28, 34, 45, 46). Faille et al. studied VWF levels before rt-PA treatment in 64 acute ischemic stroke patients (45). No association was found between VWF levels and poor outcome (mRS>2). In the study by Tóth et al. 131 consecutive acute ischemic stroke patients undergoing thrombolysis were investigated and FVIII activity and VWF levels were determined on admission, immediately after and 24 h after rt-PA treatment. FVIII levels decreased significantly immediately after lysis, that was attributed to plasmin-mediated FVIII degradation. In this study, VWF levels at all investigated time points and FVIII levels on admission and 24 h after thrombolysis were associated with worse imaging results (24 h post-lysis ASPECTS scores). In a binary backward logistic regression analysis elevated FVIII and VWF levels after thrombolysis were independently associated with poor long-term functional outcomes as defined by mRS>2 (Table 2). No association was found between FVIII levels during thrombolysis and post-lysis ICH. Navalkele et al. tested FVIII levels of 29 acute ischemic stroke patients on arrival, at 6 h and followed by 12-h intervals up to 72 h. (46) Similarly to previous findings, a significant decrease in median FVIII level from baseline to 6 h after thrombolysis was found. Baseline FVIII level and change in FVIII levels were not associated with recanalization or sICH. In another study including 74 acute ischemic stroke patients by Bembenek et al. FVIII levels on admission, at 24 h and 3 months did not differ significantly in patients with favorable or poor outcome (mRS>2) (34). To summarize these findings, elevated FVIII, and VWF levels after thrombolysis treatment might have a potential prognostic value regarding poor outcomes, but more studies are warranted on this topic.

### Factor XIII (FXIII)

Activated factor XIII (FXIIIa) cross-links fibrin chains in the last step of the clotting cascade. FXIIIa plays a crucial role in protecting the fibrin clot against prompt fibrinolysis not only via fibrin cross-linking but also by cross-linking α2-plasmin inhibitor (α2-PI) and perhaps other plasma components to the fibrin clot, thus effectively hindering its proteolysis by plasmin (17). The association of FXIII levels with thrombolysis outcomes in ischemic stroke patients has been studied in several papers (20, 32, 33, 41, 47, 48). Although it might be hypothesized that a decrease in FXIII level could be a predictor of post-lysis ICH, no such association was found in any of the investigated study cohorts. In 3 studies, a decrease of FXIII level was observed after thrombolytic treatment but it was not related to hemorrhagic complications (20, 47, 48).

In an early multicenter study investigating 63 acute ischemic stroke patients receiving intravenous thrombolysis, FXIII levels on admission were not related to poor outcomes (mRS>2) (33). In the study by Sun et al. investigating a panel of hemostasis markers in 80 acute stroke patients treated with rt-PA, FXIII activity as measured on admission, at 2 h and 24 h post-lysis was found to have no significant predictive value on recanalization or poor functional outcome (32). In the study by Schroeder et al. investigating 66 acute ischemic stroke patients, it was suggested that the decrease in FXIII levels post-stroke might be associated with an unfavorable short-term outcome and the authors expressed the need for larger studies investigating FXIII as a candidate prognostic marker (47). In a most recent study by our group FXIII levels were investigated on admission, immediately after and 24 h after thrombolysis in 132 consecutive patients with acute ischemic stroke (48). In a backward multiple regression analysis it was revealed that a FXIII level in the lowest quartile 24 h after thrombolysis is an independent predictor of short-term mortality (14 days). However, as found by previous studies, FXIII level was not a predictor of long-term outcome or mortality at 3 months. Major FXIII-A and FXIII-B polymorphisms had no impact on therapeutic outcomes.

To conclude, in the above mentioned studies, although interesting trends were seen on low FXIII levels post-event and short-term outcomes, FXIII levels showed no association with unfavorable outcomes at 3 months after the event and were not associated with post-lysis ICH.

### Global Markers of Coagulation Activation (Thrombin Generation, Thrombin-Antithrombin Complex)

Thrombin generation test is a global hemostasis assay that provides information on the speed and the amount of generated thrombin in plasma. The assay in its present form is a relatively new test but it is a potentially promising laboratory tool to elucidate coagulation mechanisms in various clinical conditions (49). Due to this fact the first studies on thrombin generation in ischemic stroke patients treated with intravenous thrombolysis were published in the past few years (23, 34, 50, 51) and patient outcomes were only assessed in two papers (23, 34). In an early study investigating thrombin generation in stroke patients, significantly higher peak thrombin concentration was found as compared to controls, but unfortunately in this study therapy outcomes were not investigated (50). In the study by Balogun et al. thrombin generation was assessed in 154 ischemic stroke patients of which 57 were treated with rt-PA (51). The authors concluded that endogen thrombin potential (ETP) or peak thrombin concentrations were not different between stroke subtypes or as compared to healthy controls, but results were not correlated with outcomes. Ninety five patients who suffered acute ischemic stroke were studied by Goldman et al. (52). Seventy one patients recevied intravenous thrombolysis, the remaining 24 served as control patients. Thrombolysed patients had markedly decreased thrombin generation parameters measured after 24 h, with the strongest impact on lag time compared to the baseline values, but again, results were not correlated with outcomes.

Our group investigated the thrombin generation test in 120 consecutive acute ischemic stroke patients before the administration of intravenous thrombolysis (23). We found that symptomatic intracranial hemorrhage was significantly associated with low ETP and peak thrombin levels (Table 1). Moreover, in a multiple logistic regression analysis it was shown that a low ETP result is an independent predictor of mortality within the first 2 weeks (OR: 6.03; 95%CI: 1.2–30.16, *p* < 0.05) and 3 months after the event (OR: 5.28; 95%CI: 1.27–21.86, *p* < 0.05) (Table 2). In the studied patient cohort, ETP was not significantly lower in those patients who had worse functional outcomes (mRS 2-5) but survived by the end of the 3rd month. In the study by Bembenek et al. it was found that a higher peak thrombin result at baseline is significantly associated with poor outcome at 3 months post-lysis (34).

Based on these interesting observations, one can conclude that the thrombin generation test might serve in the future as a useful tool to predict outcomes and safety of thrombolysis treatment, but future prospective studies involving large cohorts of patients are needed.

Antithrombin is a major inhibitor of thrombin, forming a 1:1 stable complex with it (thrombin-antithrombin complex: TAT). TAT complex is a sensitive marker for the activation of intravascular coagulation, thus useful for the risk assessment and diagnosis of thromboembolic events (53). Levels of TAT has been investigated in the population of the NINDS trial from samples collected at baseline, at 2 h after treatment and after 24 h post-lysis (27). TAT levels peaked at 2 h post-treatment selectively in the rt-PA treatment group. Increased levels of TAT (in the entire cohort) were associated with higher mortality at the third month of the follow-up that could have been due to resistance to recanalization (Table 2).

The association between pretreatment TAT levels and the outcome of thrombolysis was assessed in an interesting study by Fernandez-Cadenas et al. as well (54). TAT levels of 89 patients with middle cerebral artery occlusion were measured before the administration of rt-PA and the results correlated with the outcomes. No association was found between the measured TAT concentrations and the occurrence of hemorrhagic transformation. Decreased levels of TAT showed a significant association with better recanalization rates at all time-points (1 h: OR: 24.8, 95% CI 1.4–434.8, *p* = 0.028; 2 h: OR: 6.3 95% CI 1.5–27, *p* = 0.014; 6 h: OR: 6.4 95% CI 1.5–26.5, *p* = 0.011) after adjustment for stroke risk factors. Nevertheless, as compared to the study of Tanne, statistically significant correlation wasn't found between pre-thrombolysis levels of TAT and mortality rates.

## Fibrinolytic Parameters/Markers of Fibrinolysis Associated With Thrombolysis Outcomes

### Thrombin-Activatable Fibrinolysis Inhibitor (TAFI), Plasminogen Activator Inhibitor-1 (PAI-1)

Endogenous fibrinolysis inhibitors (e.g., TAFI, PAI-1) play an important role in the balance of coagulation and fibrinolysis and in theory may be involved in the hemorrhagic transformation after thrombolysis. After its activation TAFI removes lysine residues from partially degraded fibrin thus suppresses fibrinolysis (55). PAI-1 is the main inhibitor of t-PA: by forming stable complexes with t-PA it effectively blocks plasminogen activation (56). Moreover, it has been proposed that t-PA facilitates the disruption of BBB and PAI-1 could prevent t-PA-induced neuronal degeneration and BBB impairment (57, 58). Thus, the potential role of PAI-1 in preventing post-lysis hemorrhagic complications is intriguing.

In a prospective, longitudinal, multicenter, observational study Alessi et al. investigated the correlation between consumption of TAFI, activated/inactivated TAFI (TAFIa/ai) and the severity and outcome of stroke (59). Two groups of 109 stroke patients were enrolled in the study: one treated (68 patients) and one untreated with rt-PA (41 patients), furthermore there was a reference group of 20 patients without stroke. TAFI levels were sequentially measured in treated and not treated patients. On admission, patients had higher level of TAFIa/ai than the healthy reference group matched for age and gender. TAFIa/ai levels significantly increased at the end of thrombolysis that lasted up to 4 h. Higher levels of TAFIa/ai showed an association with a more severe day 2 NIHSS score and an unfavorable mRS score at day.

The authors concluded that their data demonstrate a considerable relationship between TAFI levels and early clinical severity during thrombolysis.

The aim of a study by Fernandez-Cadenas et al. was to evaluate whether the presence of two relatively common functional polymorphisms of the PAI-1 and TAFI genes (influencing PAI-1 and TAFI levels) have impact on recanalization rates of the middle cerebral artery among stroke patients treated with rt-PA (60). One hundred and thirty nine patients were enrolled in the study who all underwent the classic post-stroke diagnostic check-up (Doppler sonography, echocardiography, long-term ECG monitoring, complete blood cell count, and special coagulation tests) and the severity of the stroke was determined using NIHSS score. Occlusion and recanalization was diagnosed by the means of transcranial Doppler. There was no association between PAI-1 4G/5G polymorphism and the rate of recanalization, on the other hand, TAFI Thr325Ile polymorphism was significantly associated with recanalization resistance. The combination of the two polymorphisms doubled the risk of recanalization failure (OR: 11.1; 95% CI: 1.4–89.8%, *p* = 0.025).

Kim et al. also aimed to analyze pretreatment fibrinolysis inhibitor levels in patients who underwent intravenous thrombolysis and to investigate their potential association with thrombolysis failure (61). Forty three stroke patients were enrolled in the study: 17 patients were treated with intravenous t-PA, 11 with intra-arterial urokinase, and 15 with combined intravenous t-PA and intra-arterial urokinase. Patients were categorized into 2 groups according to recanalization (*n* = 30) or non-recanalization (*n* = 13), and a group of healthy volunteers (*n* = 34) were used as controls. It was found that plasma PAI-1 levels were increased in patients with acute stroke, and the increased pretreatment plasma PAI-1 levels were associated with the failure of the thrombolysis based on post-lysis angiography. TAFI levels did not differ among the groups of this cohort.

Admission PAI-1 and TAFI levels of consecutive rt-PA treated stroke patients with middle cerebral artery occlusion were studied by Ribo et al. (24). Seventy seven patients were involved in the study who had middle cerebral artery occlusion proved by transcranial Doppler. Patients with hemorrhagic transformation had lower baseline PAI-1 and higher TAFI levels. The combination of admission PAI <21.4 ng/mL and TAFI>180% had a sensitivity of 75% and a specificity of 97.6% predicting symptomatic intracranial hemorrhage (Table 1), indicating the need of further studies testing whether these biomarkers could be useful to improve the safety of thrombolysis.

### α_2_-Plasmin Inhibitor (α_2_-PI)

α_2_-PI is one of the most important regulators of fibrinolysis. It has an inhibitory effect on the fibrinolytic pathway by three ways: it forms a complex with plasmin; inhibits the binding of plasminogen to fibrin; and makes fibrin more resistant toward the effect of plasmin via its own cross-linking to fibrin mediated by FXIIIa (62).

The levels of α_2_-PI were found to correlate well with the rate of recanalization in ischemic stroke patients treated with rt-PA (33). In this study by Marti-Fabregas et al., α_2_-PI level was proved to be an independent predictor of recanalization, although it didn't have a relation with long-term outcome. In studies by others, although a decrease in α_2_-PI levels post-lysis was found, α_2_-PI levels showed no association with the occurrence of hemorrhagic transformation or poor outcomes (20, 32, 41).

### Fibrin(ogen) Degradation Products (FDPs) and D-Dimer

The *in vivo* formation of cross-linked fibrin and its subsequent secondary fibrinolytic digestion yields to a variety of soluble cross-linked FDPs. One of these products is known as D-dimer (63). It has been proved by many clinical studies that D-dimer is a valuable marker of coagulation activation and fibrinolysis (64, 65).

An increase in FDP levels 2 h post-lysis was found to be a predictor of post-lysis ICH in two studies. In an early study by Trouillas et al. FDPs were studied in 157 patients of the Lyon rt-PA trial in order to determine whether early fibrin(ogen) degradation might be indicative of hemorrhagic lesions (25). It was found that an FDP level at 2 h was a strong predictive factor for early parenchymal hematomas. An increase of FDP level >200 mg/L at 2 h post-lysis multiplied the probability of parenchymal hematoma by 4.95 (95%CI: 1.09–22.4) (Table 1). Based on the results of this single-centered study it was concluded that early fibrin(ogen) degradation can be indicative of early parenchymal hematomas that may be recognized by the detection of FDPs.

Sun et al. investigated the correlations between the presence of early intracerebral hemorrhage and the post-thrombolytic changes of hemostasis parameters (20). Seventy two patients were enrolled in the study. Similarly to the study by Trouillas et al. the presence of early parenchymal hematomas were associated with an increase in FDPs at 2 h post-lysis, indicating a massive lysis of fibrin and fibrinogen (Table 1). These results suggest that measuring not only fibrinogen but also FDPs might be useful in predicting the possibility of thrombolysis-induced intracerebral hematoma in acute stroke patients. In another study of the same authors, FDP and D-dimer levels were measured before rt-PA treatment, at 2 h and 24 h after thrombolysis in 80 stroke patients, and the results were correlated with global outcome (32). As expected, FDP and D-dimer levels increased between h0 and h2 and showed a tendency to return to the initial values at 24 h post-lysis. However, none of these values were predictive of poor outcome at 3 months.

In a study by Hsu et al. plasma D-dimer levels were evaluated after the initiation of rt-PA but within 24 h of stroke onset in 159 patients (26). Plasma D-dimer levels were significantly associated with unfavorable outcome at 3 months. Elevated D-dimer level was an independent parameter of symptomatic intracerebral hemorrhage after treatment with intravenous rt-PA. After adjustment for clinical variables in the statistical model, a higher level of D-dimer remained significantly associated with an unfavorable outcome (OR 1.90, 95% CI 1.27–2.86, *p* = 0.002) and with the occurrence of symptomatic intracranial hemorrhage (OR 2.97, 95% CI 1.15–7.70, *p* = 0.025) (Tables 1, 2).

### Global Testing of Coagulation and Fibrinolysis: Thromboelastography (TEG) and Rotational Thromboelastometry (ROTEM)

Thromboelastography (TEG) and rotational thromboelastometry (ROTEM) are global viscoelasticity-based hemostasis tests of similar methodology providing information about coagulation and fibrinolysis from whole blood (66). The viscoelastic properties of blood monitored using TEG or ROTEM were found to be associated with bleeding and thrombotic predisposition in a variety of clinical conditions. According to the present research so far, however, its usefulness to detect hypercoagulability or therapy resistance might be questionable in the ischemic stroke patient population. In a study involving 72 patients with ischemic stroke and 71 healthy subjects, the ROTEM method did not seem to be able to detect a hypercoagulable state in patients with ischemic stroke as compared to controls (67). Moreover, in another study involving 171 acute ischemic stroke patients treated with rt-PA, no robust association was found between TEG results and clinical response to therapy. Baseline and post-lysis TEG data showed no association neither with rapid improvement as detected by a change of NIHSS, nor with hemorrhagic transformation (68).

Therefore, according to these results it is not proved as yet that the above listed viscoelasticity-based point-of-care tests could be useful to guide clinical decisions in acute stroke patients, nevertheless, future prospective studies involving large number of patients are needed to confirm such negative associations.

## Studies on the Structure of Thrombi

The origin of thrombi causing ischemic stroke can be traced back to either atherosclerosis, cardioembolism or, rarely, to dissection (69). As mechanical thrombectomy became more widespread it became possible to analyze thrombi derived from the site of ischemic lesions. This technique opened a new, unique and interesting field of research providing the possibility to analyze fresh pathological thrombi of acute ischemic stroke patients. The main components of the thrombi are platelets, red blood cells, white blood cells, and cholesterol crystals (70). Knowledge of the composition of these thrombi may advance our knowledge on thrombolysis failure/therapy outcomes, as differently structured thrombi respond potentially differently to thrombolytic therapy. For instance, platelet-rich thrombi seem to be more resistant to lysis by thrombolytic therapy in rat models (71).

Most recently, a consensus statement on the current knowledge and future directions on the analysis of thrombi in acute stroke patients has been published on behalf of an international group of well-recognized clinicians and scientists (72). In this consensus paper recent research, current opportunities and limitations were published, which interested readers should consult. Here we provide a brief overview of studies that investigated thrombus composition and its relation to stroke or therapy outcomes.

The first, ground breaking study demonstrating systematic histological analysis of thrombi removed from the cerebral artery network of patients with acute ischemic stroke was published by Marder et al. (70). In that report, the authors analyzed the histology of 25 thrombi retrieved from the cerebral circulation of ischemic stroke patients and correlated the findings with clinical data. They found that the retrieved thrombi had similar histological components, independent on whether they derived from cardiac or arterial sources. Nevertheless, histological examination of these thrombi indicated that none of them were similar in overall appearance, each demonstrating a distinctive pattern. Yet the components of most cerebral thrombi were remarkably similar, 75% of them showing the pattern of lightly stained fibrin and platelet areas, interspersed with deposits of nucleated cells, often with intervening collections of erythrocytes.

In the paper by Niesten et al. the composition of the 22 extracted thrombi were found to be related to stroke subtype (73). They found that 73% of the thrombi were fresh, 18% were lytic and 9% were organized. Four thrombi were red blood cell rich, 4 thrombi were platelet rich while the rest were mixed thrombi. They found that most of the examined thrombi were fresh and the ones from the large artery atherosclerosis had the highest rate of red blood cell while the cardioembolism and cryptogenic subtype had the lowest. The large artery atherosclerosis was the only subtype with red thrombi. They found no correlation between subtype of the stroke and platelet and fibrin content. There was correlation between the red blood cell component and thrombus attenuation that can improve the attenuation on plain CT, pointing out the possibility of this as a useful imaging marker of stroke management.

In a study by Simons et al. the correlation between thrombus composition and hyperdense artery sign was investigated (74). Forty stroke patients were involved of which 28 patients underwent intravenous thrombolysis prior to the mechanical thrombectomy. Besides the basic histopathologic evaluation the samples were stained with CD34 immunostain, then they were categorized into 4 phases of thrombus formation: red blood cell dominant (11 samples), red blood cell proportion equal to fibrin (11 samples), fibrin dominant (7 samples), and organized fibrin pathology (11 samples). The hyperdense artery sign was defined by a neuroradiologist as an asymmetrical, increased density in one of the intracranial arteries. In 29 cases the radiologist was able to assess hyperdense artery sign on the pre-treatment CT. The conclusion of the examination was that thrombi were composed of early phase pathology. The impact of this data is that the presence or absence of hyperdense artery sign might enable neurologists to predict the composition of the thrombus, which might provide the benefit of different treatment options. They didn't find statistically significant connection between thrombus composition and cardioembolic stroke data.

The presence of white blood cells with an emphasis on neutrophil extracellular traps (NETs) was studied in a particularly interesting paper by Laridan et al. (75). Sixty-eight thrombi were extracted from ischemic stroke patients undergoing endovascular treatment. The thrombi were immunostained and characterized for the presence of neutrophils and NETs. Both neutrophils and NETs were detected in all thrombi. Older thrombi and those of cardioembolic origin contained more NETs. *Ex vivo* lysis of thrombi indicated that adding DNase might have prothrombolytic effect, which is an interesting observation with potential future therapeutic value.

In a study by Sporns et al. involving a remarkably large cohort (*n* = 187) of patients histopathologic investigation of clots retracted with mechanical thrombectomy was used in order to find specific clot patterns that might help to differentiate between causes of ischemic stroke (76). Of the 187 patients included 77 had cardioembolic source of stroke, 35 had large artery atherosclerosis, 11 were of other origin (dissection, radiogenic stenosis, tumor associated) and 64 cases were identified as cryptogenic stroke. Erythrocyte-rich thrombi were shown to have a significant correlation with non-cardioembolic stroke sources, while cardioembolic cases showed a significant association with fibrin-rich thrombi. The immunhistochemical markers CD68/KiM1P were higher in cardioembolic cases as compared to noncardioembolic strokes. Based on this study, it was concluded that histological thrombus features vary significantly according to the underlying cause and might be used to help to differentiate between cardioembolic and non-cardioembolic stroke types.

In another study with a relatively large number of clot samples (*n* = 137) histological clot composition was also studied in the relation of ischemic stroke causes (77). In this paper the authors aimed to find specific patterns that may help to differentiate between the causes of cryptogenic stroke. They found significant differences between the composition of thrombi of cardioembolic and noncardioembolic stroke patients. Cardioembolic thrombi consisted of significantly higher rate of fibrin/platelets, more leukocytes and less erythrocytes than noncardioembolic thrombi. Thrombi received from cryptogenic stroke patients had the same basic pattern as cardioembolic thrombi, with higher proportions of fibrin/platelets and smaller fractions of red blood cells. Similarities were also found between cryptogenic and cardioembolic strokes in terms of interventional and outcome parameters.

Despite the significant achievements in the efficacy of mechanic thrombectomy, little is known why in some patients recanalization is less successful. The composition of thrombi and its relation to the outcomes after thrombectomy were studied in only few studies as yet (78–81).

In a prospective study by Boeckh-Behrens et al. thrombus composition was analyzed with respect to the etiology of stroke, recanalization and clinical outcome (78). Thirty-four patients with acute ischemic stroke were included in the study, with distal internal carotid artery/carotid-T, anterior cerebral artery or middle cerebral artery occlusion. Histopathological examination of the extracted thrombi was carried out and it was found that the higher rate of white blood cells in the thrombus was associated with extended mechanical recanalization time, less favorable recanalization and worse clinical outcome (mRS>2). The percentage of leukocytes within the thrombi and the mRS scores showed a positive correlation (*r* = 0.358, *p* = 0.057) with a borderline statistical significance. The authors found significant relation between the white blood cell fraction of the thrombi and cardioembolic etiology of stroke. These data suggest that white blood cell-mediated immunological or hemostatic processes may play an important role in the development of stroke and might have impact on outcomes.

The aim of the prospective study by Sporns was to identify the effect of thrombus composition on the time and grade of revascularization and the risk of procedure-related secondary embolisms (79). Moreover, the authors aimed to evaluate the correlation of pre-interventional CT thrombus attenuation with the histological analysis of thrombi and other outcome parameters. Samples of 180 patients with complete diagnostic and histological workup were included in the study. The cause of the stroke as determined by the TOAST-classification was arterioembolic in 34 patients, cardioembolic in 75 patients, of other determined cause in 11 patients, and cryptogenic in 60 patients. In 168 patients recanalization was complete and 27 patients (15%) suffered secondary embolism. Besides the basic histopatological examination, detailed immunohistochemistry staining was also performed in the extracted thrombi samples (CD3, CD20, and CD68/KiM1P CD3; CD20, and CD68/KiM1P). Based on their observations fibrin-rich thrombi with low erythrocyte rate had significantly longer intervention times, while thrombi with a low rate of red blood cells and low CT-density caused embolisms in the thrombectomy process more often suggesting that these thrombi have higher fragility.

Similar results were obtained in the study of Gunning et al. (80). In this paper, the relationship between clot composition and the resistance to sliding (friction) was studied, which might contribute to resistance to clot removal. They found that fibrin-rich clots with <20% red cell content have significantly higher friction coefficient that might contribute to resistance to clot removal.

Prochazka et al. investigated thrombus composition in a prospective cohort study including 131 patients with ischemic stroke (81). Recanalization was successful in 115 patients from whom 90 samples were extracted and analyzed histologically. They found a significant relationship between plasma VWF and the VWF found in the thromboembolus, platelets, or fibrin. There was a correlation between the area of immunostained VWF and platelet count, CD31- positive cells and fibrin. The amount of all CD31-positive cells correlated with the number of neutrophils in the thrombus. D-dimer levels showed a significantly positive relationship with plasma VWF levels and with long-term prognosis (mRS>2). Significant correlations were found between the numbers of NK (natural killer) cells and the fibrin content of the thrombus, blood neutrophil levels and VWF levels within the thrombus and the number of lymphocytes and fibrin content of the thrombus.

To conclude, insights into thrombus composition might reveal stroke etiology and might provide relations to clinical outcomes. Understanding thrombus composition might serve as a key in advancing our knowledge and improving acute stroke care.

## Conclusion

Despite advances in understanding the mechanisms leading to poor clinical outcomes and hemorrhagic complications following rt-PA treatment of acute ischemic stroke, prediction of individual patient risks is still unavailable. Clinical failure of thrombolysis as well as hemorrhagic complications are possibly related to hemostatic events. Data on markers of coagulation and/or fibrinolysis in acute stroke patients are numerous but most studies include relatively few patients thus they lack statistical power. Based on these data clinical recommendations regarding treatment and monitoring cannot be given as yet. Nevertheless, few markers seem to be potentially useful in predicting therapeutic outcomes. A simplified evaluation regarding the prognostic values of hemostasis markers/tests on thrombolysis safety and outcome is summarized in Table 3.

**Table 3:** A simplified evaluation regarding the prognostic values of hemostasis markers/tests on thrombolysis safety and outcome.

**Predictive marker**	**References**	**Prognostic value on**	
		**safety (ICH)**	**poor outcome**
Fibrinogen	(20–22, 25, 27, 32–35)	+	–
Fibrin clot structure	(34, 38)	–	+
FVII, FSAP	(33, 40–43)	–	–
FVIII, VWF	(28, 34, 45, 46)	–	+
FXIII	(20, 32, 33, 41, 47, 48)	–	+
Thrombin generation assay	(23, 34, 50–52)	+	+
TAT complex	(27, 54)	–	+
TAFI	(24, 59–61)	+	–
PAI-1	(24, 60, 61)	+	–
α2 plasmin inhibitor	(20, 32, 33, 41)	–	–
FDP	(20, 25, 32)	+	–
D-dimer	(20, 26, 32)	+	+
Thromboelastometry	(67, 68)	–	–

*–, no prognostic value or insufficient data; +, potential prognostic value; FDP, fibrin(ogen) degradation product; FSAP, factor seven activating protease; FVII, factor seven; FVIII, factor VIII; FXIII, factor XIII; ICH, intracerebral hemorrhage; PAI-1, plasminogen activator inhibitor-1; TAFI, thrombin-activatable fibrinolysis inhibitor; TAT complex, thrombin-antithrombin complex; VWF, von Willebrand Factor*.

Well-designed, prospective clinical studies involving large cohorts of patients are awaited to better understand the hemostatic and fibrinolytic system during rt-PA therapy and to validate the prognostic value of promising markers in clinical decision making. Conclusions of such studies might lead to an improvement of the efficacy and/or safety of thrombolysis treatment. Studies on thrombus composition also seem useful in advancing our knowledge that could ultimately provide a key to improve acute stroke therapy.

## Author Contributions

RK and NT were responsible for literature screening. ZB, IS, and LC screened literature, processed the articles and wrote the paper.

### Conflict of Interest Statement

The authors declare that the research was conducted in the absence of any commercial or financial relationships that could be construed as a potential conflict of interest.
